# A traditional formula of aconitum complex alleviates post-ischemic stroke by improving neural function: Aconitum complex alleviates post-ischemic stroke

**DOI:** 10.3724/abbs.2023291

**Published:** 2024-01-16

**Authors:** Ji Ma, Ruiqi Pu, Qinyang Zhou, Maoru Li, Jing Shang

**Affiliations:** 1 School of Life Science and Technology Kunming University of Science and Technology Kunming 650500 China; 2 School of Traditional Chinese Pharmacy Jiangsu Key Laboratory of TCM Evaluation and Translational Research China Pharmaceutical University Nanjing 211198 China; 3 College of Traditional Chinese Medicine Yunnan University of Chinese Medicine Kunming 650500 China

Stroke is the second leading cause of death worldwide. There are more than 2 million new stroke patients in China every year, and ischemic strokes account for 60%‒70% of these patients [1]. Even with proper hospital treatment, 30% of stroke patients still die within a year after diagnosis. Furthermore, more than 80% of stroke survivors suffer from motor disorders ( *e*. *g*., hemiplegia and aphasia), and approximately 15%‒30% are permanently disabled. Stroke affects not only individuals but also their families, causing severe social consequences [2].

The pathogenesis of stroke is associated with inflammation, oxidative stress, blood-brain barrier damage, apoptosis, and ultimately neuronal damage in the brain [ 3, 4]. Injury to brain nerve cells in the basal layer, thalamus and cortex can cause motor disorders and other sequelae [5]. Patients diagnosed with ischaemic stroke often need immediate thrombolysis treatment; however, this treatment restores only the blood supply to the brain, and rapid blood flow possibly leads to ischaemia-reperfusion injury in the brain. Treatment methods for post-ischemic stroke mainly focus on restoring neuron function, which is associated with the functions of different types of cells, such as astrocytes, oligodendrocytes, and microglia. Therefore, when studying the repair of neurological deficits in patients suffering from postischemic stroke, it is necessary for scholars and clinicians to take a variety of cells into account [6].

Sanwu Jiao (SW) is a traditional formula of aconitum complex originated from Yi ethnic people in Yunnan Province and dated to the Qing Dynasty. The product is composed of five different types of herbs, namely, *Aconitum* carmichaelii Debx (Chuanwu), *A*. *vilmorinianum* Kom (Huangcaowu), *Typhonium giganteum* Engl (Baifuzi), *Polygonum multiflorum* Thunb (Heshouwu), and *A*. *carmichaelii* Debx (Fupian), with a ratio of 4:16:2:3:1. There is evidence that complex formulas can effectively improve hemiplegia in post-ischemic stroke patients in northeastern Yunnan Province [7]. Although SW has shown its potential for treating post-ischemic stroke patients, there is still a lack of systematic research on this complex formula [1]. To investigate the effects and pharmacological mechanism of Sanwu Jiao in the treatment of post-ischemic stroke patients, we established a classical acute stroke rat model induced by koizumi middle cerebral artery occlusion (MCAO), which was described in previous studies [ 8, 9].

**Figure 1:**
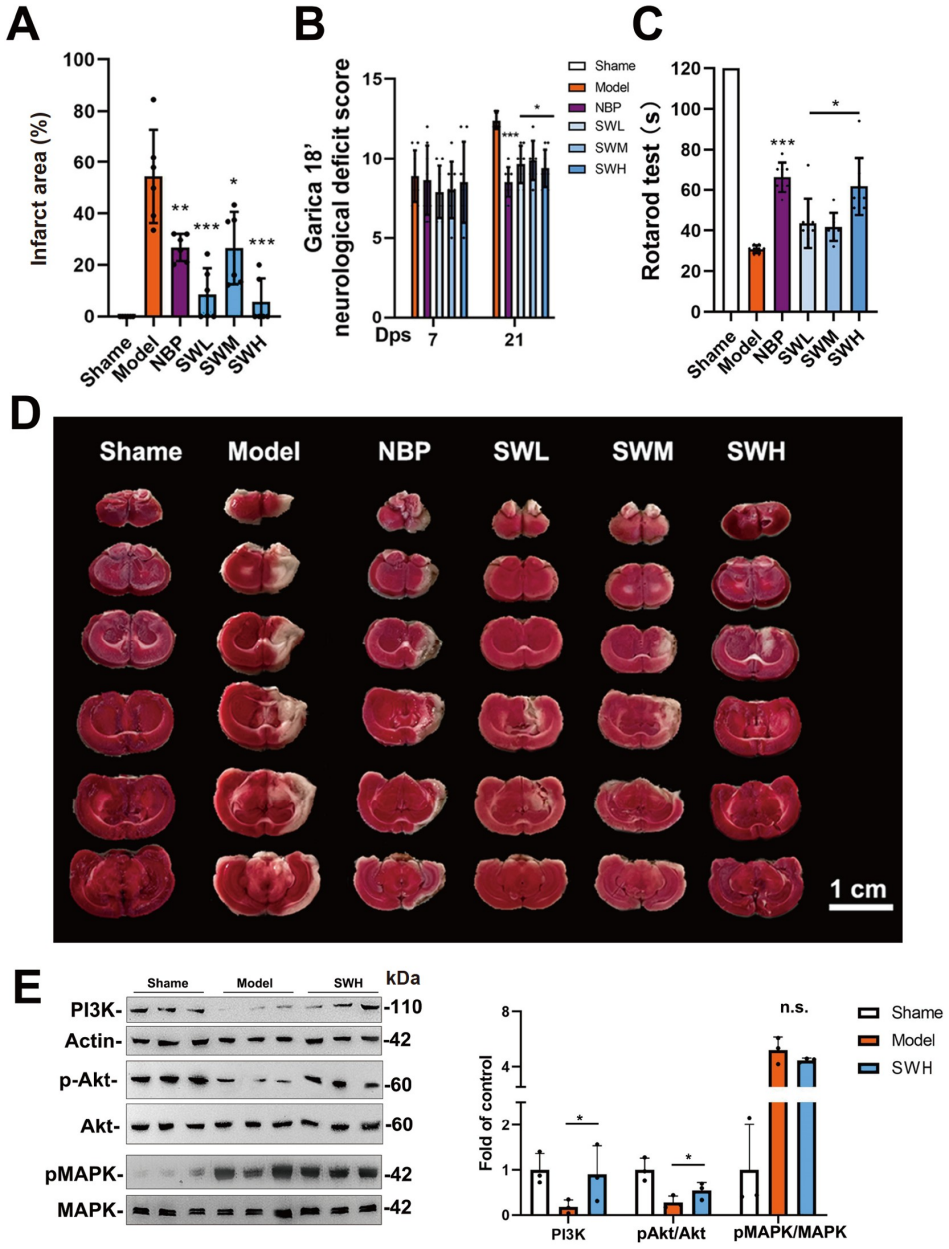
Figure 1 Effect of SW on MCAO post-ischemic stroke rat model (A) Infarcted area of the brain. (B) mNSS of MCAO post-ischemic stroke rat model. (C) Time of the rotarod test. (D) TTC staining of the cerebral infarction on the MCAO post-ischemic stroke rat model. (E) Relative expressions of PI3K, pAkt, and pMAPK in cerebral infarction. Data are shown as the mean±SD. *P<0.05, **P<0.01, ***P<0.001. Significance was calculated by ANOVA followed by Tukey’s test.

Seven days after MCAO surgery, we regrouped the surviving rats (60 of 114 rats) so that all the groups had similar modified neurological severity scores (mNSSs) before drug administration. There were five groups, namely, (i) model, (ii) NBP (54 mg/kg/day), (iii) SWL (450 mg/kg/day), (iv) SWM (900 mg/kg/day), and (v) SWH (1800 mg/kg/day). The doses of NBP and SW were determined according to the instructions for the conversion of human and rat serum. All the drugs were administered by gavage for 14 consecutive days. After two weeks of drug treatment, all three SW groups showed improvements in their mNSSs compared with those of the model group ( Figure 1B). In addition, we conducted a rotarod test to assess motor balance in the rats. As shown in Figure 1C, the three SW groups and the NBP group exhibited significant improvements in motor dysfunction.

In the post-ischemic stroke rat model established by MCAO, edema and necrosis of the striatum and cortical regions were examined by TTC staining ( Figure 1D). We used 3-butylphthalide (NBP), a commercially available drug for the treatment of moderate stroke, as a positive control drug. In the NBP group, brain edema and necrotic regions were significantly improved according to the TTC results ( Figure 1D). Notably, cerebral edema was improved to various degrees in both the cerebral cortex and the striatum region in all three SW groups. TTC staining results further demonstrated that SW also improved cerebral infarction ( Figure 1A,D).

**Figure 2:**
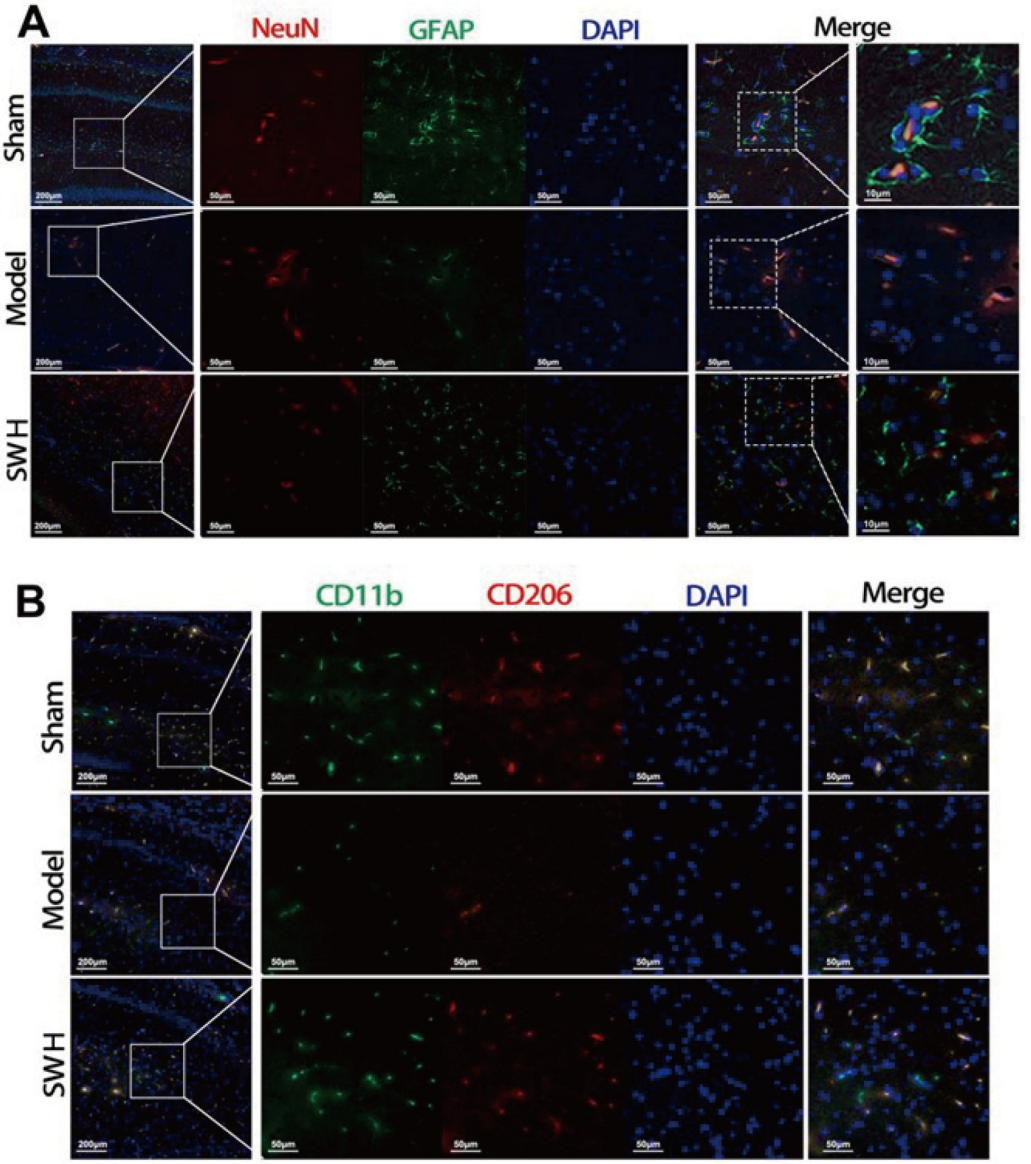
Figure 2 Immunofluorescence microscopy of cerebral infarction on MCAO post-ischemic stroke rat model (A) Immunofluorescence of neurons (NeuN) and neuroglia (GFAP). (B) Immunofluorescence of microglia (CD11b) and M2 type microglia (CD206).

To further investigate neuronal and neuroglial injuries, we performed an immunofluorescence experiment on the MCAO-induced post-ischemic stroke rat model with cerebral infarction. The NeuN protein and glial fibrillary acidic protein (GFAP) were tested to evaluate the activity of neurons and neuroglia. As illustrated in Figure 2A, in the sham group, normal neurons were surrounded by active GFAPs. However, there was no GFAP expression around the neurons in the model group. Furthermore, GFAP expression in the 1800 mg/kg SW/day group was considerably greater than that in the model group, suggesting that the neuroglial injury was ameliorated by SW. We also used CD11b, which is an important immune cell in nerves, as a marker of microglia and used CD206 to further detect whether M2 microglia are activated. The results showed that some of the microglia in the sham group were type M2 ( Figure 2B). On the other hand, the expression levels of CD11b and CD206 were decreased in the model group but significantly increased in the SW group. These findings suggested that SW activated M2 microglia and restored microglia. Despite these relevant findings, the mechanism underlying the activation of M2 microglia cells by SW awaited further analysis.

The PI3K and MAPK pathways are known to regulate the apoptosis and regeneration of cells [ 10, 11]. According to related literature, SW plays a regulatory role in the MCAO-induced post-ischemic stroke rat model through the PI3K and MAPK pathways. Therefore, we performed western blot analysis to detected PI3K expression and phosphorylation of Akt and MAPK. Compared with those in the model group, PI3K expression and Akt phosphorylation were significantly increased in the group treated with the same dose of SW as in the immunofluorescence experiment ( Figure 1E). Meanwhile, SW slightly inhibited MAPK phosphorylation. These results further demonstrated that SW affects the downstream PI3K and MAPK pathways.

In summary, the results of this study demonstrated that the traditional aconitum complex drug SW improved cerebral infarction and neurobehavioral deficits in the MCAO-induced post-ischemic stroke rat model, promoted the activation of M2 microglia, restored the expression of microglia and neuroglial cells, and activated the downstream PI3K and Akt phosphorylation pathways. Although it remains to be further clarified whether different substances in SW have neuroprotective effects on various targets, our findings sufficiently demonstrate that SW alleviates the consequences of postischemic stroke by improving neural function.
